# Prevalence of Pathological Germline Mutations of *hMLH1* and *hMSH2* Genes in Colorectal Cancer

**DOI:** 10.1371/journal.pone.0051240

**Published:** 2013-03-19

**Authors:** Dandan Li, Fulan Hu, Fan Wang, Binbin Cui, Xinshu Dong, Wencui Zhang, Chunqing Lin, Xia Li, Da Wang, Yashuang Zhao

**Affiliations:** 1 Department of Epidemiology, Public Health College, Harbin Medical University, Harbin, Heilongjiang Province, P. R. China; 2 Department of Abdominal Surgery, The Tumor Hospital of Harbin Medical University, Harbin, Heilongjiang Province, P. R. China; 3 Department of Science and Technology Administration, Harbin Medical University, Harbin, Heilongjiang Province, P. R. China; Ohio State University Medical Center, United States of America

## Abstract

The prevalence of pathological germline mutations in colorectal cancer has been widely studied, as germline mutations in the DNA mismatch repair genes *hMLH1* and *hMSH2* confer a high risk of colorectal cancer. However, because the sample size and population of previous studies are very different from each other, the conclusions still remain controversial. In this paper, Databases such as PubMed were applied to search for related papers. The data were imported into Comprehensive Meta-Analysis V2, which was used to estimate the weighted prevalence of *hMLH1* and *hMSH2* pathological mutations and compare the differences of prevalence among different family histories, ethnicities and related factors. This study collected and utilized data from 102 papers. In the Amsterdam-criteria positive group, the prevalence of pathological germline mutations of the *hMLH1* and *hMSH2* genes was 28.55% (95%CI 26.04%–31.19%) and 19.41% (95%CI 15.88%–23.51%), respectively, and the prevalence of germline mutations in *hMLH1*/*hMSH2* was 15.44%/10.02%, 20.43%/13.26% and 15.43%/11.70% in Asian, American multiethnic and European/Australian populations, respectively. Substitution mutations accounted for the largest proportion of germline mutations (*hMLH1*: 52.34%, *hMSH*2: 43.25%). The total prevalence of mutations of *hMLH1* and *hMSH2* in Amsterdam-criteria positive, Amsterdam-criteria negative and sporadic colorectal cancers was around 45%, 25% and 15%, respectively, and there were no obvious differences in the prevalence of germline mutations among different ethnicities.

## Introduction

Colorectal cancer (CRC) is a major worldwide public health problem [1], and is the second leading cause of cancer death in developed countries. In developing countries, CRC represents the sixth or seventh leading cause of cancer death [2].

It is estimated that hereditary nonpolyposis colorectal cancer (HNPCC) accounts for somewhere between less than 1% to 13% [3], [4] of colorectal cancers, which make it the most common inherited CRC syndrome [5], [6]. HNPCC is characterized by an autosomal dominant inheritance pattern of early onset colorectal cancer, which is associated with extra colonic malignancies, such as endometrial, urological and upper gastrointestinal cancers [7]. There is no characteristic phenotype associated with HNPCC, and its diagnosis is dependent on the recognition of a strong family history suggestive of dominant inheritance [8].

HNPCC, also known as Lynch syndrome (LS), is caused by a germline mutation in the DNA mismatch repair (MMR) genes [9], [10]. A normal functioning MMR system can recognize and correct the base-pair mismatches and small nucleotide (1–4 base pair) insertion/deletion mutations, which is essential for the maintenance of genomic stability [11].

There are at least of five germline mutations in the DNA mismatch repair genes that can cause Lynch syndrome, including *hMLH1*, *hMSH2*, *hMSH6*, *hPMS1* and *hPMS2* [12]–[17]. Mutations in *hMLH1* and *hMSH2* account for the majority of case of Lynch syndrome [18].

The *hMSH2* gene, which is a component of the DNA mismatch repair pathway, was the first gene identified to be associated with HNPCC. It serves as the “scout” that recognizes and binds directly to the mismatched DNA sequence [19], [20] and can form a heterodimer with *hMSH6* when a single base-pair mismatch is recognized or with *hMSH3* if two to eight nucleotide insertions or deletions exist [11].

The *hMLH1* gene protein product is also a component of the DNA mismatch repair pathway, which has been shown to form a heterodimer with the *hMLH3*, *hPMS2* and *hPMS1* genes. However, this protein has unknown enzymatic activity and likely acts as a “molecular matchmaker” that recruits other DNA repair proteins to the mismatch repair complex [21].

Since the *hMLH1* and *hMSH2* genes were found in humans, the prevalence of germline mutations has been widely studied not only in case of colorectal cancer with a suggestive family history but also in sporadic colorectal cancer. However, the results of these studies are inconsistent because the sample sizes were small, and the ethnic backgrounds were varied [22]–[24]. Therefore, a systematic review and meta-analysis is essential to provide recommendations for genetic tests based on family history and a basis for the prevention, early diagnosis and treatment of colorectal cancer.

## Methods

### 1. Search strategy and selection criteria

Databases, including PubMed, Embase and Cochrane Library, were applied to search for related papers published from January 1993 to March 2011 with the following keywords: *hMLH1*, *hMSH2*, mutation, hereditary nonpolyposis colorectal cancer, colorectal cancer and/or carcinoma, tumor or neoplasm. Chosen papers were limited to those that were published in English and fulfilled the following selection criteria: 1) paper assessing only a specific type of mutation or only specific regions of genes were excluded; 2) the mutations had to be germline mutations with pathological features but not somatic, studies that revealed somatic alteration of the MMR genes presence were excluded; 3) case reports were excluded; 4) repetitive reports were unified by using the latest or the largest edition; 5) research on polymorphism was excluded; 6) Lynch syndrome patients with known MMR gene mutations were excluded; 7) the detection patient was limited to a diagnosis of colorectal cancer rather than other Lynch syndrome related cancer such as endometrial cancer. The specific process of study selection has been shown in Figure S4 in supporting information.

### 2. Classification of family history and ethnicity

We categorized colorectal cancer patients who met the stringent Amsterdam criteria (I or II) [25] as the Amsterdam-criteria positive group (AC+). Patients without any family history of cancer, regardless of the onset age, were categorized in the sporadic cancer group. Others who had a family history but did not strictly conform to the Amsterdam criteria were defined as the Amsterdam-criteria negative group (AC−). Additionally, we named the patients with an ambiguous family history or those who did not have enough information to be re-classified again, as the family history not clear group.

Because the information about ethnicity of patients was not well-defined, we had to define the ethnicity based on continents, including Asian, American multiethnic, European/Australian or mixed ethnicities (some studies did not offer this type of data or this data included American, European and Australian).

### 3. MSI status and category

If more than 30% of the typically used microsatellite markers show instability, the tumor will be considered MSI-high. MSI-stable (MSS) is defined as no markers indicating instability [26], [27]. Otherwise, the tumor is defined as MSI-low. For patients without information about microsatellite status, we classify them as MSI-not identified. Additionally we define studies that combined MSI-high and MSI-low tumors as MSI.

### 4. Determination of pathogenicity

We determined the pathogenicity of mutations primarily by three methods combined. First, we deferred to the interpretations of the original papers and the pathogenic definition including: a frameshift mutation that would be predicted to result in a truncated protein; nonsense mutations; missense mutations ascertained with a functional assay; large genomic deletions that removed at least one exon; or duplication of exon, to segregation of the alteration with cancer in the kindred [28]. Second, we used the analytic program PolyPhen to predict this mutation to be pathogenic [29], If PolyPhen score>2.0 then the change was predicted to affect protein function. Last, we checked two websites including “International Society for Gastrointestinal Hereditary Tumours Incorporated (InSiGHT) (www.insight-group.org/mutations/)” and “MMR Gene Unclassified Variants Database (www.mmruv.info)” to further determine pathogenicity. To apply functional assays will be more accurate and objective when testing missense variants for pathogenicity. But many articles could not do this due to various limitations; some studies distinguished between pathological changes with polymorphism or determined pathogenicity when the same variants founding in the control population. In the InSiGHT database, its “Reported pathogenicity” was categorized as reported pathogenic or probably pathogenic and “Concluded pathogenicity” was unknown. We then considered it as reported pathogenicity according to these results. In our Meta-analysis, we categorized those reported pathogenic mutations or probably pathogenic meeting the definition as pathogenic mutations.

### 5. Data Extraction

Two investigators (Dandan Li and Fulan Hu) independently extracted data and checked all of the differences in the variables until an agreement was reached on all items. Information such as the first author, published years, continent, country, family history, mutation sites, mutation types, and MSI phenotype and detection methods was collected from each article.

### 6. Statistical analysis

Data were imported into Comprehensive Meta-Analysis V2, which estimated the weighted prevalence and compared the difference of prevalence among related factors. A significant α level of 0.05 was applied. For multiple tests, an α level of 0.05 was adjusted to α divided by the number of multiple tests. Heterogeneity between studies was assessed with meta-regression and I^2^ statistics. I^2^ statistics included 25, 50 and 75 corresponding to low, medium and high heterogeneity, respectively [30]. If I^2^ was ≤50 combined with the characteristics of the data [31], the fixed-effects model was used. Otherwise, random-effects models were adopted. The publication bias was assessed visually using a funnel plot. The rank correlation method suggested by Begg [32] and the linear regression approach proposed by Egger et al. [33], [34] were used to quantitatively analyze the potential publication bias.

## Results

After filtering for potentially relevant citation, there were 796 abstracts retrieved. We then excluded those studies that had no clear gene mutation detection data. Finally, a total of 279 articles on *hMLH1* and *hMSH2* germline mutations in colorectal cancer were searched in an electronic database. However, there were only 102 papers included in this study [6], [8], [24], [26], [35]–[132] based on the selection criteria. A clear family history was provided in 82 of these papers. The detected population came from Asian, American, European/Australian and mixed ethnic populations in 22, 11, 63 and 6 papers, respectively. Basic characteristics of the included articles are shown in Table S1 in supporting information.

### 1. The prevalence of germline pathological mutations in different family histories

In total, 861 of 7057 and 698 of 7096 colorectal cancer cases reported had *hMLH1* and *hMSH2* gene mutations, respectively. Additionally, 1526 of 6965 cases had a mutation in one gene or the other when both genes were screened.

The highest prevalence of pathological germline mutations occurred in the AC+ group and was 28.55% (95%CI 26.04%–31.19%) and 19.41% (95%CI 15.88%–23.51%) in the *hMLH1* and *hMSH2* genes (*P* = 0.00<0.05), respectively. The prevalence in the AC− group was 16.70% (95%CI 14.53%–19.13%) and 11.30% (95%CI 9.49%–13.42%) (*P* = 0.00<0.05), respectively. In the sporadic cancer group, the prevalence of mutations was 8.72% (95%CI 6.12%–12.29%) and 7.28% (95%CI 5.12%–10.26%) (*P* = 0.47>0.05) (Table 1). High heterogeneity among the all included studies was observed in both *hMLH1* (I^2^ = 80.10%) and *hMSH2* (I^2^ = 79.98%) across all colorectal cancers. However, the heterogeneity was at an acceptable level for both genes in the three subgroups with clear family histories (Table 1).

**Table 1:** Prevalence of *hMLH1* and *hMSH2* gene germline mutation in different family history and ethnicity.

Family history	Ethnicity	*hMLH1*							*hMSH2*						
		N@	Detected cases	Mutation cases	Prevalence (%) and 95%CI	Range of prevalence (%)	*P* for Z test	I^2^ (%)	N@	Detected cases	Mutation cases	Prevalence (%) and 95%CI	Range of prevalence (%)	*P* for Z test	I^2^ (%)
AC+#	Asian	16	256	65	27.98(22.53–34.18)	0.00–50.00	0.00	0.00	15	244	32	17.56(9.93–29.14)	0.00–75.00	0.00	55.87
	American multiethnic	9	172	53	31.17(17.31–49.50)	0.00–100.00	0.04	72.70	9	172	35	19.54(8.46–38.97)	0.00–75.00	0.00	72.90
	European/Australian	44	844	211	25.64(20.89–31.05)	0.00–77.14	0.00	54.48	42	852	137	19.14(16.33–22.31)	0.00–77.78	0.00	45.83
	Mixed population*	3	131	43	32.94(25.42–41.45)	25.00–34.92	0.00	0.00	3	131	44	33.78(26.16–42.35)	25.00–38.10	0.00	0.00
AC−	Asian	11	313	43	16.81(12.69–21.93)	0.00–40.00	0.00	49.60	11	313	30	12.18(5.84–23.65)	0.00–63.64	0.00	69.93
	American multiethnic	5	70	8	17.35(8.80–31.37)	0.00–40.00	0.00	48.22	5	70	6	10.93(5.27–21.30)	0.00–20.00	0.00	0.00
	European/Australian	34	881	116	16.69(14.09–19.66)	0.00–50.00	0.00	31.14	32	925	76	10.33(8.32–12.75)	0.00–50.00	0.00	14.51
	Mixed population*	2	27	4	14.88(5.70–33.60)	12.50–15.79	0.00	0.00	2	27	5	20.60(1.51–81.41)	5.26–50.00	0.34	81.38
Sporadic colorectal cancer	Asian	6	439	7	3.21(0.88–11.03)	0.00–14.81	0.00	63.65	6	439	9	3.64(1.96–6.65)	0.00–7.41	0.00	23.43
	American multiethnic	5	60	4	10.28(4.28–22.70)	0.00–22.22	0.00	0.00	5	60	2	5.89(2.08–15.61)	0.00–10.00	0.00	0.00
	European/Australian	12	214	9	7.47(4.06–13.34)	0.00–100.00	0.00	13.41	11	213	10	7.58(4.05–13.76)	0.00–80.00	0.00	46.44
	Mixed population*	3	36	6	16.71(7.70–32.53)	14.29–17.65	0.00	0.00	3	36	7	21.90(11.04–38.79)	0.00–25.00	0.00	0.00
Family history not clear	Asian	3	154	21	11.30(3.79–29.14)	0.00–20.45	0.00	63.03	3	154	11	8.45(2.99–21.63)	2.27–14.29	0.00	65.30
	American multiethnic	1	32	4	12.50(4.77–28.94)	12.50	0.00	0.00	1	32	9	28.13(15.33–45.82)	28.13	0.02	0.00
	European/Australian	14	3247	260	8.69(5.17–14.24)	0.00–20.48	0.00	92.19	14	3247	280	8.31(4.99–13.53)	0.00–36.36	0.00	91.92
	Mixed population*	2	181	7	3.87(1.85–7.89)	3.85–3.92	0.00	0.00	2	181	5	2.85(1.19–6.67)	2.31–3.92	0.00	0.00
Asian Subtotal	22	1162	136	15.44(11.49–20.45)	0.00–50.00	0.00	58.72	21	1150	82	10.02(6.15–15.92)	0.00–75.00	0.00	76.14	
American multiethnic Subtotal	11	334	69	20.43(12.11–32.34)	0.00–100.00	0.00	74.09	11	334	52	13.26(7.26–22.99)	0.00–42.86	0.00	70.46	
European/Australian Subtotal	62	5186	596	15.43(12.50–18.89)	0.00–100.00	0.00	83.23	60	5237	503	11.70(9.37–14.51)	0.00–80.00	0.00	79.77	
Mixed population Subtotal*	6	375	60	15.02(7.19–28.75)	3.85–34.92	0.00	84.48	6	375	61	14.39(6.13–30.22)	0.00–50.00	0.00	87.23	
Total	101	7057	861	15.74(13.40–18.41)	0.00–100.00	0.00	80.10	98	7096	698	11.74(9.74–14.07)	0.00–80.00	0.00	79.98	

* This type of population include American, European and Australian.

@ This indicates the number of studies have been included.

# We categorized colorectal cancer patients who met the stringent Amsterdam criteria as Amsterdam-criteria positive group (AC+). Others have a strong family history but not strictly in conformity with Amsterdam criteria were defined as Amsterdam-criteria negative group (AC−).

The total prevalence of the two genes' pathological mutations in papers that detected both of them were 44.70% (95%CI 39.13%–50.40%), 24.65% (95%CI 20.37%–29.50%), 11.56% (95%CI 7.11%–18.23%) and 17.02% (95%CI 11.24%–24.93%) in the AC+, AC−, sporadic cancer and family history not clear groups, respectively (*P* = 0.00<0.05) (Table 2).

**Table 2:** Total prevalence of germline mutation of *hMLH1 & hMSH2* genes in different family history and ethnicity.

		Both detected					
Family history	Ethnicity	Detected cases	Mutation cases	Prevalence(%) and 95%CI	Range of prevalence (%)	*P* for Z test	I^2^ (%)
AC+	Asian	244	93	38.01 (31.90–44.53)	18.75–100.00	0.00	16.83
	American multiethnic	172	88	54.02 (33.72–73.07)	23.08–100.00	0.71	80.49
	European/Australian	807	328	42.59 (35.56–49.93)	0.00–100.00	0.05	67.94
	Mixed population*	131	87	66.09 (57.44–73.80)	50.00–73.02	0.02	44.62
AC−	Asian	313	73	27.07 (15.94–42.08)	0.00–80.00	0.00	75.98
	American multiethnic	70	14	22.86 (13.76–35.49)	8.33–40.00	0.01	38.38
	European/Australian	839	184	23.65 (18.94–29.12)	0.00–60.00	0.00	54.20
	Mixed population*	27	9	38.59 (9.48–79.04)	21.05–62.50	0.61	74.69
Sporadic colorectal cancer	Asian	439	16	5.31 (1.79–14.76)	0.00–22.22	0.00	74.21
	American multiethnic	60	6	12.84 (6.00–25.37)	0.00–22.22	0.00	0.00
	European/Australian	213	18	12.13 (5.80–23.65)	0.00–80.00	0.00	54.46
	Mixed population*	36	13	37.63 (23.10–54.80)	14.29–41.67	0.16	0.00
Family history not clear	Asian	154	32	21.04 (15.28–28.26)	14.29–22.73	0.00	0.00
	American multiethnic	32	13	40.63 (25.26–58.08)	40.63	0.29	0.00
	European/Australian	3247	540	17.19 (10.23–27.42)	0.00–54.55	0.00	96.39
	Mixed population*	181	12	6.67 (3.83–11.38)	6.15–7.84	0.00	0.00
Asian Subtotal		1150	214	24.95 (18.51–32.74)	0.00–100.00	0.00	77.76
American multiethnic Subtotal		334	121	34.77 (22.39–49.64)	0.00–100.00	0.05	81.91
European/Australian Subtotal		5106	1070	27.51 (22.65–32.98)	0.00–100.00	0.00	90.48
Mixed population Subtotal*		375	121	30.84 (12.06–59.19)	6.15–73.02	0.18	94.46
Total		6965	1526	27.89 (23.94–32.21)	0.00–100.00	0.00	89.19

Multiple comparisons among four group, a value was 0.007 with two-tailed.

* This type of population include American, European and Australian.

### 2. The prevalence of pathological germline mutations in different ethnicities

In the *hMLH1* gene, the prevalence of pathological germline mutations in the AC+ group ranged from 25.64% to 32.94% in the four ethnicities evaluated (*P* = 0.48>0.05). In the AC− group, they ranged from 14.88% to 17.35% (*P* = 0.99>0.05), and they ranged from 3.21% to 16.71% (*P* = 0.13>0.05) in the sporadic cancer group (Table 1).

In the *hMSH2* gene, the mutation prevalence ranged from 17.56% to 33.78% in the AC+ group, from 10.33% to 20.60% in the AC− group and from 3.64% to 21.90% in the sporadic cancer group (AC+: *P* = 0.00<0.05; AC−: *P* = 0.91>0.05; sporadic: *P* = 0.00<0.05) in the four ethnicities evaluated. In the AC+ and sporadic cancer group, differences were seen in the mixed ethnicities group compared to the European/Australian group (*P* = 0.000<0.007) and in the Asian group compared to the mixed ethnicities group (*P* = 0.000<0.007), respectively (Table 1).

Refers to the articles that had both gene that were detected, in the AC+ group, the total mutation prevalence of *hMLH1* and *hMSH2* for Asian, American multiethnic, European/Australian and mixed ethnicities was 38.01%, 54.02%, 42.59% and 66.09%, respectively (Table 2). In the AC− group, the prevalence was around 25% (*P* = 0.83>0.05). In sporadic cases, there was a wide range and difference in the prevalence (from 5.31% to 37.63%, *P* = 0.00<0.05) (Table 2). There were obvious differences among these ethnicities in the AC+ and sporadic cancer groups (all had *P* = 0.000<0.007). Further analysis showed that these differences were seen in Asian compared to mixed ethnicities and European/Australian compared to mixed ethnicities. No differences were observed among the three clear ethnicities.

### 3. The mutation distribution in different exons

All of the exons in these two genes showed mutations. The highest mutation prevalence of 3.62% was found in exon 16 of the *hMLH1* gene, with 2.19 mutations/100 bp, which, remarkably, accounted for 16.36% of all mutations. In addition to exon 16, the prevalence of mutation was higher in exon 2, exon 6, exon 8, exon 12, exon 13 and exon 19. Mutations in these seven exons (including exon16) accounted for 55.45% of the total mutations. In the *hMSH2* gene, the mutation prevalence and densities in different exons were generally lower than those in the *hMLH1* gene. The highest prevalence of mutation was 2.62% in exon 7. Those in exon 3, exon 5, exon 11 and exon 12 were also higher than in other exons. The total mutations in these five exons accounted for 53.39% of the total mutations (Table 3).

**Table 3:** Prevalence of *hMLH1* and *hMSH2* gene germline mutation in different exons.

	*hMLH1*						*hMSH2*					
Exon	Length (b p)	Detected cases	Mutation cases	Prevalence of Mutation (%) and 95% CI	Mutation Density (/100 bp)	Component Ratio (%)	Length (b p)	Detected cases	Mutation cases	Prevalence of Mutation (%) and 95% CI	Mutation density (/100 bp)	Component Ratio (%)
Exon1	176	4942	39	2.05 (1.62–2.59)	1.17	7.09	279	4905	15	1.60 (1.21–2.11)	0.58	3.50
Exon2	91	4942	31	2.10 (1.64–2.68)	2.31	5.64	155	4905	6	1.47 (1.09–1.98)	0.95	1.40
Exon3	99	4942	15	1.60 (1.21–2.12)	1.62	2.73	279	4905	41	2.27 (1.79–2.28)	0.82	9.56
Exon4	74	4942	29	1.92 (1.50–2.45)	2.59	5.27	147	4905	20	1.65 (1.26–2.15)	1.12	4.66
Exon5	73	4942	4	1.55 (1.14–2.09)	2.12	0.73	150	4905	36	2.14 (1.68–2.71)	1.42	8.39
Exon6	92	4942	22	2.24 (1.72–2.91)	2.44	4.00	134	4905	23	2.05 (1.57–2.67)	1.53	5.36
Exon7	43	4942	7	1.58 (1.18–2.13)	3.68	1.27	200	4905	64	2.62 (2.13–3.23)	1.31	14.92
Exon8	89	4942	33	2.16 (1.68–2.77)	2.43	6.00	110	4905	19	1.63 (1.24–2.14)	1.48	4.43
Exon9	113	4942	12	1.89 (1.42–2.51)	1.67	2.18	124	4905	20	1.89 (1.45–2.46)	1.52	4.66
Exon10	94	4942	20	1.72 (1.32–2.25)	1.83	3.64	151	4905	13	1.60 (1.20–2.14)	1.06	3.03
Exon11	154	4942	29	1.98 (1.54–2.55)	1.29	5.27	98	4905	31	2.07 (1.60–2.66)	2.11	7.23
Exon12	371	4942	33	2.29 (1.79–2.93)	0.62	6.00	246	4905	57	2.30 (1.85–2.85)	0.93	13.29
Exon13	149	4942	57	3.26 (2.65–4.01)	2.19	10.36	205	4905	36	1.77 (1.39–2.26)	0.87	8.39
Exon14	109	4942	18	1.91 (1.46–2.50)	1.75	3.27	248	4905	18	1.66 (1.26–2.18)	0.67	4.20
Exon15	64	4942	17	1.66 (1.27–2.17)	2.59	3.09	176	4905	27	2.03 (1.56–2.64)	1.15	6.29
Exon16	165	4942	90	3.62 (3.00–4.35)	2.19	16.36	443	4905	3	1.52 (1.11–2.06)	0.34	0.70
Exon17	93	4942	26	1.95 (1.51–2.53)	2.10	4.73	—	—	—	—	—	—
Exon18	114	4942	29	2.09 (1.64–2.67)	1.83	5.27	—	—	—	—	—	—
Exon19	361	4942	39	2.58 (2.03–3.28)	0.72	7.09	—	—	—	—	—	—

### 4. The mutation types

As shown in Table 4, there were three main types of gene mutations, including substitutions (with inclusion of transition and transversion), deletions or insertions and large genomic rearrangements. Substitution accounted for 60.97% and 53.77% of all three point mutations in *hMLH1* and *hMSH2* gene, respectively. The next highest was deletion, which accounted for 24.15% and 36.98% of the total, respectively.

**Table 4:** Prevalence of *hMLH1* and *hMSH2* gene germline mutation by types, family histories and ethnicities.

		*hMLH1* (%)					*hMSH2* (%)				
	Category	Deletion	Insertion	Substitution	Large genomic rearrangement	Not identified	Deletion	Insertion	Substitution	Large genomic rearrangement	Not identified
Ethnicity	Asian	3.13(13.56)	6.09(22.03)	9.85(55.93)	3.90(5.93)	2.21(2.54)	3.88(30.77)	2.93(6.41)	6.92(52.56)	2.65(10.26)	1.50(0.00)
	American multiethnic	4.99(21.05)	4.15(10.53)	12.03(50.88)	2.35(3.51)	6.63(14.04)	6.90(34.62)	3.53(7.69)	7.07(21.15)	3.21(23.08)	5.79(13.46)
	European/										
Australian	3.79(21.88)	3.03(10.85)	8.88(52.44)	2.49(13.92)	1.74(0.90)	4.23(30.39)	1.81(7.33)	5.73(44.40)	2.84(15.30)	2.50(2.59)	
	Mixed population	4.90(23.81)	3.41(14.29)	7.13(46.03)	2.90(15.87)	1.04(0.00)	4.47(20.00)	3.16(9.23)	7.45(41.54)	5.16(29.23)	1.04(0.00)
Family history	AC+	7.17(18.13)	6.60(11.90)	16.61(52.41)	9.05(14.45)	4.50(3.12)	8.06(28.63)	4.19(8.06)	11.34(41.13)	7.48(17.74)	4.43(4.44)
	AC−	5.27(22.67)	3.98(10.47)	11.01(55.23)	4.49(9.88)	3.18(1.74)	4.73(24.37)	3.17(5.88)	6.92(44.54)	4.12(19.33)	3.76(5.88)
	Sporadic	2.75(3.85)	3.26(7.69)	6.45(61.54)	3.58(19.23)	3.27(7.69)	3.63(32.14)	2.92(3.57)	4.82(39.29)	4.27(25.00)	2.75(0.00)
	Not clear	2.34(25.00)	1.41(16.25)	4.57(49.17)	1.28(9.58)	0.71(0.00)	3.12(32.95)	1.43(7.95)	4.11(45.08)	1.86(13.64)	0.83(0.38)

Component ratio in parentheses.

### 5. MSI status and prevalence of germline mutations

In MSI-high phenotype, the mutation prevalence in the *hMLH1* gene was 29.84% (95%CI 22.43%–38.48%), 22.03% (95%CI 13.66%–33.53%) and 18.34% (95%CI 9.39%–32.72%) in AC+, AC− and sporadic cancer, respectively. The next highest mutation prevalence was in the MSI-low and MSS groups. There were no statistical differences among different family histories in all of these three MSI phenotype categories (*P* was 0.25, 0.41 and 0.93, respectively).

The mutation prevalence in the *hMSH2* gene was also the highest in AC+ (26.81% 95%CI 19.02%–36.35%), followed by AC− (24.84%, 95%CI 16.14%–36.21%) and sporadic cancer (7.46%, 95%CI 2.64%–19.34%) with MSI-high phenotype, with marginal statistical differences (*P* = 0.04<0.05). Cases with MSS manifested the lowest prevalence of mutations in the different family history group (*P* = 0.48>0.05). The prevalence in MSI-low was moderate (*P* = 0.98>0.05) (Table 5).

**Table 5:** MSI phenotype and prevalence of *hMLH1* and *hMSH2* gene germline mutation.

		*hMLH1* *				*hMSH2* #				*hMLH1 & hMSH2* both detected			
MSI Status	Family history	Detected cases	Mutation cases	Prevalence of Mutation (%) and 95%CI	I^2^ (%)	Detected cases	Mutation cases	Prevalence of Mutation (%) and 95%CI	I^2^ (%)	Detected cases	Mutation cases	Prevalence of Mutation (%) and 95%CI	I^2^ (%)
MSI-High	AC+	138	38	29.84(22.43–38.48)	0	138	30	26.81(19.02–36.35)	28.48	138	68	53.41(38.02–68.17)	54.22
	AC−	91	15	22.03(13.66–33.53)	0	91	19	24.84(16.14–36.21)	15.53	91	34	38.80(27.87–50.98)	41.75
	Sporadic	45	8	18.34(9.39–32.72)	0	45	2	7.46(2.64–19.34)	0	45	10	22.54(12.55–37.11)	0
	Not clear	132	13	11.80(5.22–24.54)	50.70	132	21	19.67(13.10–28.46)	48.42	132	34	28.03(14.30–47.62)	73.89
	Subtotal	406	74	20.85(16.88–25.47)	0	406	72	21.21(17.11–25.98)	33.10	406	146	37.82(30.38–45.91)	50.54
MSI-Low	AC+	5	0	16.67(0.95–80.64)	0	5	0	16.67(0.95–80.64)	0	5	0	16.67(0.95–80.64)	0
	AC−	15	2	20.95(6.16–51.69)	0	15	1	12.68(2.55–44.67)	0	15	3	29.92(10.79–60.10)	0
	Sporadic	3	0	12.50(0.73–73.44)	0	3	0	12.50(0.73–73.44)	0	3	0	12.50(0.73–73.44)	0
	Not clear	31	0	3.03(0.43–18.62)	0	31	1	5.06(1.02–21.64)	0	31	1	5.06(1.02–21.64)	0
	Subtotal	54	2	12.17(4.82–27.49)	0	54	2	9.18(3.46–22.17)	0	54	4	16.11(7.21–32.19)	0
MSI▵	AC+	151	51	35.80(28.36–43.99)	39.58	151	49	35.98(28.59–44.10)	23.57	151	100	61.77(41.44–78.68)	73.94
	AC−	47	13	27.25(16.43–41.64)	24.66	47	10	24.73(15.01–37.95)	0	47	23	46.14(33,26–59.56)	16.98
	Sporadic	119	6	9.87(4.83–19.12)	4.04	119	15	16.71(6.66–36.05)	57.17	119	21	23.33(10.40–44.38)	65.00
	Not clear	232	35	14.75(8.54–24.29)	50.44	232	19	7.41(4.19–12.79)	15.80	232	54	19.99(11.41–32.64)	66.43
	Subtotal	549	105	17.84(12.46–24.89)	63.24	549	93	16.95(10.96–25.28)	70.31	549	198	34.05(22.37–48.04)	85.86
MSS	AC+	48	2	11.35(4.87–24.27)	0	48	2	11.35(4.87–24.27)	0	48	4	13.94(6.47–27.49)	0
	AC−	102	2	5.55(2.42–12.21)	0	102	1	5.47(2.29–12.51)	0	102	3	6.12(2.77–12.99)	0
	Sporadic	29	0	4.86(0.98–20.90)	0	29	1	7.46(1.86–25.59)	0	29	1	7.46(1.86–25.59)	0
	Not clear	72	0	0.69(0.04–10.14)	0	72	0	0.69(0.04–10.14)	0	72	0	0.69(0.04–10.14)	0
	Subtotal	251	4	4.70(2.42–8.93)	0	251	4	4.74(2.39–9.17)	0	251	8	6.52(3.64–11.40)	1.96
MSI not identified	AC+	1017	267	26.85(22.16–32.13)	57.32	1013	158	18.06(13.92–23.09)	56.96	968	401	43.78(37.10–50.69)	69.51
	AC−	953	133	17.88(15.30–20.78)	32.87	997	80	10.59(8.58–13.00)	40.90	911	205	24.77(19.38–31.07)	62.58
	Sporadic	492	11	7.42(4.38–12.29)	37.04	491	9	5.82(3.36–9.91)	36.94	491	19	7.35(3.16–16.15)	66.78
	Not clear	3335	265	7.71(4.38–13.23)	93.82	3335	280	7.45(4.31–12.57)	93.49	3335	545	15.49(8.78–25.85)	97.04
	Subtotal	5797	676	16.16(13.13–19.72)	83.83	5836	527	11.10(8.78–13.96)	82.09	5705	1170	27.78(22.82–33.34)	90.77

* Compared among these five subgroups in *hMLH1* gene, *P* value (2-sided) = 0.00.

# Compared among these five subgroups in *hMSH2* gene, *P* value (2-sided) = 0.00.

▵ MSI refers to the cases can not identify MSI-high or MSI-low.

In articles that detected both genes, the prevalence of mutation was the highest in AC+ (53.41%, 95%CI 38.02%–68.17%), followed by AC− (38.80%, 95%CI 27.87%–50.98%) and sporadic cancer (22.54%, 95%CI 12.55%–37.11%) with MSI-high phenotype (*P* = 0.02<0.05). These were followed by cases with MSI-low and MSS in the different family history group (*P*>0.05).

### 6. Prevalence of germline mutations in different subject select setting

There were 28 population-based series articles and 29 clinic-based series articles that were evaluated in this study. In the *hMLH1* gene, the mutation prevalence was 12.49 (95%CI 8.65–17.71) in the population-based group and 17.39 (95%CI 13.62–21.93) in the clinic-based group (*P* = 0.13). In the *hMSH2* gene, the mutation prevalence were 10.50% (95%CI 6.94%–15.59%) and 12.03% (95%CI 8.47%–16.80%), respectively (*P* = 0.62) (Table S5 in supporting information). To further consider the difference in each family history group, there were no significant statistics in any group.

### 7. Prevalence of *hMLH1* and *hMSH2* gene intron area germline mutation

Our results found that the highest intronic mutation frequency in the AC+ group was 8.49% (95%CI 6.43%–11.13%) and 5.42% (95%CI 3.82%–7.64%) in the *hMLH1* and *hMSH2* genes, respectively. The prevalence in the AC− group was 4.15% (95%CI 2.75%–6.23%) and 4.01% (95%CI 2.57%–6.21%), respectively. In sporadic cancer, the prevalence of mutations was 5.81% (95%CI 3.04%–10.84%) and 5.51% (95%CI 2.76%–10.68%), respectively (Table S6 in supporting information). And there were no differences between different ethnicities in either gene (*hMLH1*: *P* = 0.78 in AC+, *P* = 0.12 in AC−, *P* = 0.38 in sporadic cancer; *hMSH2*: *P* = 0.44 in AC+, *P* = 0.41 in AC−, *P* = 0.58 in sporadic cancer) (Table S6 in supporting information).

### 8. Publication bias

Funnel plots of the prevalence of pathological mutation in these two genes both in general and with different family histories showed some extent of asymmetry, with small studies on the left side of the plot (Figure S1, S2 and S3 in supporting information). Detailed results of an Egger regression, a Begg correlation and a “Trim and Fill” analysis for different family histories with these two genes, both separately and together, are shown in Table 6.

**Table 6:** Egger' regression, Begg and Mazumdar rank correlation and Trim & Fill results.

	*hMLH1*							*hMSH2*							*hMLH1&hMSH2*						
	Linear regression (two tailed)		Rank correlation (two tailed)		Trim and fill			Linear regression (two tailed)		Rank correlation (two tailed)		Trim and fill			Linear regression (two tailed)		Rank correlation (two tailed)		Trim and fill		
Category	Intercept	*P* value	Tau	*P* value	Studies filled	Observed values (%)	Adjusted values (%)	Intercept	*P* value	Tau	*P* value	Studies filled	Observed values (%)	Adjusted values (%)	Intercept	*P* value	Tau	*P* value	Studies filled	Observed values (%)	Adjusted values (%)
AC+	−1.02	0.01	−0.17	0.03	22	28.55	33.94	−1.32	0.00	−0.10	0.23	12	19.41	23.00	0.32	0.59	0.12	0.14	0	44.70	44.70
AC−	−1.08	0.00	−0.20	0.03	15	16.70	19.45	−0.44	0.35	−0.06	0.55	12	11.30	14.26	−0.35	0.50	−0.01	0.89	1	24.65	24.96
Sporadic	−2.50	0.00	−0.18	0.19	10	8.72	13.94	−0.92	0.22	0.14	0.33	9	7.28	10.76	−1.38	0.16	−0.09	0.53	6	11.56	16.75
Not clear	−1.78	0.13	−0.26	0.11	9	8.47	15.96	−1.79	0.11	−0.23	0.16	3	8.21	10.41	−2.18	0.22	−0.15	0.35	0	17.02	17.02
Total	−0.47	0.29	−0.21	0.00	1	15.74	15.94	−0.81	0.046	−0.16	0.02	22	11.73	15.48	0.17	0.77	−0.07	0.30	0	27.89	27.89

## Discussion

Based on a systemic review and meta-analysis, we found that the total mutation prevalence of *hMLH1* and *hMSH2* in patients having both genes screened was 44.70%, 24.65% and 11.56% in the AC+, AC− and sporadic cancer groups, respectively. However, the reported mutations in these two genes were very different in Lynch syndrome [6], [48], [59]. One reason for the difference was that we limited the mutation region to exons and the mutation type to pathogenicity, which allowed us to provide more stable mutation prevalence results by executing a systematic review and meta-analysis.

Although papers on mutations in different ethnicities have been published, no reports have explicitly described any differences among them. Our analysis found that there was no substantial statistical difference between these four ethnicities with different family histories across both genes, either separately or together.

Although InSiGHT database have collected information about new mutations in different exons, few papers or websites provided information on exon-specific prevalence and detailed mutation types. Our results showed that a remarkable high prevalence of mutation occurred in exon 16. It was also noteworthy that the mutations in exon 16 and exon 2 were mainly aggregated at c.1852_1854delAAG and c.199G>A, which accounted for 37.78% and 29.03% of the mutations, respectively (data not shown). In *hMSH2*, the highest mutation prevalence was found in exon 7. The total mutations in exon 3, exon 5, exon 7, exon 11 and exon 12 amounted to 53.39% of the total (Table 3). Therefore, when performing *hMLH1* and *hMSH2* gene mutation tests, it would be important to focus attention on these exons and their common mutation points.

In Wei W. et al. [133], three mutations (exon 8 c.649C*>*T, exon 14 c.1625A*>*T and exon 15 c.1721T*>*C) in *hMLH1* and four mutations (exon 1 c.23C*>*T and c.187dupG, exon 3 c.505A*>*G and exon 7 c.1168C*>*T) in *hMSH*2 had a much higher prevalence in Asian populations than in European populations. Furthermore, three mutations (exon 13 c.1453G*>*C, exon 16 c.1742C*>*T and c.1758dupC) in *hMLH1* and two (exon 7 c.1255C*>*A and exon 12 c.1886A*>*G) in *hMSH2* were only found in the Asian population, which implies that specific mutations in this population should be highlighted when screening for mutations in these two genes.

Our results showed that the major mutation type of both genes was substitution and deletion. The substitution of a nucleotide could result in missense, nonsense and silent mutations, while deletion and insertion typically lead to frameshift. There were no differences in mutation type by ethnicity in *hMSH2* (deletion *P* = 0.18>0.05; insertion *P* = 0.11>0.05; substitution *P* = 0.85>0.05), but there were in the *hMLH1* gene. For insertion, differences existed between the Asian and European/Australian populations (*P* = 0.00<0.05). Insertion mutations accounted for a larger proportion in the Asian population than in the European/Australian population (Table 4 and Table S4 in supporting information).

These results suggested that not only point mutations occurred frequently in colorectal cancer but also large genomic rearrangements were present to some extent. Initially, the detection methods for large genomic rearrangements were mainly southern analysis [115] and conversion analysis [18]. Recently, more sensitive MLPA analysis was performed for patients who had no point mutations to determine the occurrence of large genomic deletions of these two genes [134]. In our 102 studies, there were only five papers using MLPA separately or combined with other methods, representing 4.90% of the total studies. The prevalence of large genomic rearrangements in *hMLH1* and *hMSH2* was 6.76% (95%CI 3.11%–14.05%) and 13.56 (95%CI 11.19%–16.32%) (Data not shown), respectively, which was higher than the results in Table 4, where the subjects and detection methods were not specified. Therefore, the mutation prevalence in future results is expected to be higher with the use of more sensitive methods to identify large genomic deletions.

Studies have revealed that cases with negative microsatellite instability may also carry germline mutations. The mutation prevalence is widely ranged in different MSI situations and with different family histories [70]. The prevalence of mutation was 53.41% in AC+ patients' with tumors exhibiting MSI-high phenotype, which suggested that the predicted value of MSI-high for mutations in these two genes was 53.41% in the AC+ group. The next highest was 38.80% in the AC− group and then 22.54% in the sporadic group. If we took MSI as one group (combined MSI-high, MSI-low and MSI (cannot identify MSI-high or MSI-low)), the corresponding predicted value was 57.12% (95%CI 50.43%–63.55%) in the AC+ group (Table 5).

Several techniques for detecting mutations are commonly used, including immunohistochemistry followed by DNA-sequencing, single-strand conformational polymorphism followed by DNA-sequencing, heteroduplex analysis followed by DNA-sequencing, denaturing gradient gel electrophoresis followed by DNA-sequencing, denaturing high-performance liquid chromatography followed by DNA-sequencing, and direct DNA-sequencing. Analysis of the effect of different detection methods on the prevalence have found that, in general, there was no significant difference in prevalence detected by the four methods in AC+ (*P* = 0.60>0.05) and AC− (*P* = 0.30>0.05) group. In the sporadic group, there were too few studies to analysis (Table S2 in supporting information).

A distinction between a population-based and clinic-based series was made, for this was a potentially important bias of the analysis. However, we observed that there were no significant differences in the mutation prevalence between the clinic-based and population-based groups in either gene. When considering the effects of family history, the conclusion did not change (Table S5 in supporting information).

There was higher heterogeneity in total prevalence. Meta-regression results showed that 22.34% of this heterogeneity was explained by different family histories (*P* = 0.00) (Table S3 in supporting information). Subgroup analysis showed that the heterogeneity was at moderate or low levels for different family histories with respect to the prevalence in these two genes. In addition to family history, factors such as years since publication, different ethnicities and detection methods were also analyzed, and none of them showed any statistically significant effect on heterogeneity (Table S3 in supporting information).

From funnel plots (Figure S2 and S3 in supporting information), we observed that the figures from the meta-analysis for the mutation prevalence with different family histories of the two genes were all skewed to the left. Further quantity analysis by Egger regression and Begg correlation methods on the extent of asymmetry found that the left-side asymmetry was statistically significant in some situations (Table 6). This indicated that an increased number of accepted publications and small study sizes had no effect on positive results. This only illustrated that more studies with small sample sizes and lower detection ability were conducted and published. However, we can still use “Trim and Fill” to adjust the value of the original data [135]. We observed that the adjusted value increased from 28.55% to 33.94% in the *hMLH1* gene and from 19.41% to 23.00% in the *hMSH2* gene in the AC+ group (Table 6).

In addition to mutations in exons, some of most common pathogenic variants are intronic. So, we systematically searched papers on associations between intervening sequence or intron area mutations and Lynch syndrome, and we also analyzed the difference of prevalence among related factors such as family history and ethnicities (Table S6 in supporting information). When we calculated the two genes combined, the intronic mutation frequency was 12.30% (95%CI 9.80%–15.33%) in the AC+ group and 5.90% (95%CI 4.08%–8.47%) in the AC− group. Moreover, we found that the most common pathogenic deleterious variants in Lynch syndrome were *hMSH2* Intron5 c.942+3A>T, and *hMLH1* Intron9 c.790+1G>A, with a mutational consequence of deletion of exon5 in *hMSH2* and a deletion of exon9–10 in *hMLH1*.

Sensitivity analysis indicated that the results of our study were reliable and stable. However, this meta-analysis still has some limitations, such as the family history information of the patients not being clearly explained and studies providing insufficient ethnicity information having to be roughly classified. In the sporadic colorectal cancer group, the detection population was usually filtered by MSI phenotype or onset age [64], [129]. Moreover, insufficient studies on sporadic colorecal cancer and information on gender need to be further analyzed. In addition, in order to control the quality and uniform standards for articles, the written language was limited to English, which may have affected the number of included studies.

Not only *hMLH1* and *hMSH2* gene, we remained concerned about the prevalence of these mutations of other MMR genes, in particular *hMSH6* or *hPMS2*. In 2009, a systematic review was conducted and a meta-analysis was undertaken to determine the frequency of *hMSH6* mutation in colorectal and endometrial cancers by our academic team [136]. As to *hPMS2* gene, mutations in *hPMS2* gene are a rare cause of Lynch syndrome [15], [16]. And the mutation frequency were currently less than 2% (http://www.med.mun.ca/mmrvariants), moreover, there was fewer papers study *hPMS2* gene mutation [137], so we did not include it avoiding destabilizing results. In spite of these limitations, our results are still reliable and yield important conclusions. Data on sporadic cases were not sufficient or detailed, and, hence, large, well-designed studies with information on ethnicity, gender and age of onset are needed.

### Data statement

We declare that all the data analyzed in this paper were extracted from the on-line published articles and we take the responsibility for the integrity of the data and the accuracy of the data analysis.

## Supporting Information

Table S1 **Characteristics of included studies about weighted prevalence of** ***hMLH1*** **and** ***hMSH2*** **germline mutation in colorectal cancer.**
(DOC)

Table S2 **Prevalence of mutation of** ***hMLH1 & hMSH2*** **genes both detected with different detection methods.**
(DOC)

Table S3 **Meta-regression result by different variance.**
(DOC)

Table S4 **Prevalence of** ***hMLH1*** **and** ***hMSH2*** **gene germline mutation by types in detail.**
(DOC)

Table S5 **Prevalence of** ***hMLH1*** **and** ***hMSH2*** **gene germline mutation by clinic and population-based.**
(DOC)

Table S6 **Prevalence of** ***hMLH1*** **and** ***hMSH2*** **gene intron area germline mutation in different family history and ethnicity.**
(DOC)

Figure S1 **Funnel plot for meta-analysis of prevalence of** ***hMLH1*** **(left)/** ***hMSH2*** **(right) gene germline mutation in total colorectal cancer.**
(DOC)

Figure S2 **The funnel plot of prevalence of** ***hMLH1*** **gene mutation in colorectal cancer.**
(DOC)

Figure S3 **The funnel plot of prevalence of** ***hMSH2*** **gene mutation in colorectal cancer.**
(DOC)

Figure S4 **Process of study selection.**
(DOC)
