# Supplementary material for: Prevalence of Pathological Germline Mutations of hMLH1 and hMSH2 Genes in Colorectal Cancer
Source: PLoS One. 2013 Mar 19;8(3):e51240. doi: 10.1371/journal.pone.0051240 (PMC3602519; doi:10.1371/journal.pone.0051240)
Supplement: Table S1 — Characteristics of included studies about weighted prevalence of hMLH1 and hMSH2 germline mutation in colorectal cancer. (DOC) [file pone.0051240.s001.doc]

**Table S1 Characteristics of included studies about weighted prevalence of *hMLH1* and *hMSH2*** germline mutation in colorectal cancer

|  |  |  |  | *hMLH1* | | *hMSH2* | |
| --- | --- | --- | --- | --- | --- | --- | --- |
| First author | Year | Race (country) | Detected cases | Mutation cases | Prevalence of Mutation (%) and 95%CI | Mutation cases | Prevalence of Mutation (%) and 95%CI |
| Jae-Gahb Park | 1999 | Asian(Korea) | 88 | 18 | 20.45(13.28-30.15) | 2 | 2.27(0.57-8.63) |
| Seung-Yong Jeong^ | 2003 | Asian (Korea) | 230 | 0 | 0.22(0.01-3.36) | 1 | 0.43(0.06-3.02) |
| Seon Ae Roh | 2003 | Asian(Korea) | 21 | 1 | 4.76(0.67-27.14) | 0 | 2.27(0.14-27.74) |
| Young-Kyoung Shin | 2004 | Asian(Korea) | 164 | 28 | 17.07(12.06-23.62) | 5 | 3.05(1.27-7.11) |
| Mariapina Montera | 2006 | Asian (Korea) | 54 | 3 | 5.56(1.80-15.86) | 3 | 5.56(1.80-15.86) |
| Siu Tsan Yuen | 2002 | Asian (China) | 41 | 9^§^ | 21.95(11.84-37.07) | 9 | 21.95(11.84-37.07) |
| Shan-Run Liu | 2004 | Asian (China) | 28 | 5 | 17.86(7.63-36.38) | 2 | 7.14(1.79-24.48) |
| Long Cui | 2004 | Asian(China) | 12 | 3^§^ | 25.00(8.28-55.18) | 3^§^ | 25.00(8.28-55.18) |
| Yuan Ying | 2004 | Asian(China) | 63 | 17 | 26.98(17.48-39.20) | 2 | 3.17(0.80-11.82) |
| Ding-Cun Luo | 2005 | Asian(China) | 5 | 1 | 20.00(2.72-69.10) | 1 | 20.00(2.72-69.10) |
| Xu-Lin Wang | 2006 | Asian(China) | 31 | 3 | 9.68(3.15-26.06) | 4 | 12.90(4.93-29.74) |
| Jian Qiu Sheng | 2006 | Asian(China) | 21 | 6 | 28.57(13.43-50.76) | 2 | 9.52(2.39-31.13) |
| Chao-Fu Wang | 2007 | Asian(China) | 12 | 4 | 33.33(13.09-62.41) | — | — |
| Hei-Ying Jin | 2008 | Asian(China) | 35 | 0 | 13.90(0.09-18.67) | 5 | 14.29(6.07-30.05) |
| Chang-Hua Zhang | 2008 | Asian(China) | 5 | 1 | 20.00(2.72-69.10) | 1 | 20.00(2.72-69.10) |
| Hong-Li Yan^ | 2008 | Asian(China) | 167 | 12 | 7.19(4.13-12.23) | 9 | 5.39(2.83-10.03) |
| J.Q. Sheng | 2008 | Asian(China) | 76 | 17 | 22.37(14.38-33.07) | 11 | 14.47(8.20-24.28) |
| S-C Wei | 2003 | Asian (China, Taiwan) | 15 | 3 | 10.00(6.59-46.98) | 0 | 3.13(0.19-35.03) |
| M. Miyaki | 1995 | Asian (Japan) | 11 | 2 | 18.18(4.58-50.70) | 5 | 45.45(20.28-73.19) |
| Yun-Qing Bai | 1999 | Asian (Japan) | 37 | 0 | 1.32(0.08-17.84) | 9 | 24.32(13.17-40.52) |
| Yukihiro Abe | 2000 | Asian (Japan) | 31 | 0 | 1.56(0.10-20.60) | 0 | 1.56(0.10-20.60) |
| Sachio Nomura | 2000 | Asian (Japan) | 15 | 3 | 20.00(6.59-46.98) | 8 | 53.33(29.30-75.91) |
| Benedito Mauro Rossi | 2002 | American (Brazilian) | 25 | 7 | 28.00(13.97-48.22) | 2 | 8.00(2.01-26.94) |
| Carlos Sarroca | 2000 | American (Uruguay) | 19 | 13 | 68.42(45.16-85.08) | 0 | 2.50(0.15-29.81) |
| Alejandro Giraldo | 2005 | American (Columbia) | 23 | 9 | 39.13(21.77-59.76) | 1 | 4.35(0.61-25.22) |
| Anna L. Millar | 1999 | American (Canada) | 40 | 1 | 2.50(0.35-15.73) | 6 | 15.00(6.90-29.59) |
| Klaus K.F. Herfarth | 1997 | American (USA) | 12 | 1 | 8.33(1.16-41.32) | 0 | 3.85(0.24-40.32) |
| Thomas K. Weber | 1997 | American (USA) | 32 | 5^§^ | 15.63(6.66-32.47) | 3 | 9.38(3.06-25.35) |
| Sapna Syngal | 1999 | American (USA) | 70 | 7 | 10.00(4.84-19.52) | 4 | 5.71(2.16-14.26) |
| Thomas K. Weber | 1999 | American(USA) | 11 | 2 | 18.18(4.58-50.70) | 1 | 9.09(1.26-43.86) |
| David J. Peel | 2000 | American(USA) | 11 | 2 | 18.18(4.58-50.70) | 3 | 27.27(9.05-58.57) |
| Jonathan P. Terdiman | 2001 | American(USA) | 32 | 4 | 12.50(4.77-28.94) | 9 | 28.13(15.33-45.82) |
| Anja Wagner | 2003 | American(USA) | 59 | 18 | 30.51(20.14-43.31) | 23 | 38.98(27.46-51.88) |
| Alessandra Viel | 1997 | European (Italy) | 17 | 4 | 23.53(9.12-48.55) | 1 | 5.88(0.82-32.03) |
| Valeria Pensotti | 1997 | European(Italy) | 14 | 3 | 21.43(7.07-49.43) | 3 | 21.43(7.07-49.43) |
| Maurizio Genuardi | 1998 | European (Italy) | 32 | 3 | 9.38(3.06-25.35) | 3 | 9.38(3.06-25.35) |
| M Ponz de Leon | 1999 | European (Italy) | 36 | 1 | 2.78(0.39-17.26) | 1 | 2.78(0.39-17.26) |
| Maria Cristina Curia | 1999 | European (Italy) | 30 | 5 | 16.67(7.11-34.32) | 5 | 16.67(7.11-34.32) |
| C Ghimenti | 1999 | European (Italy) | 6 | 1 | 16.67(2.28-63.13) | 0 | 7.14(0.43-57.72) |
| D. Calistri | 2000 | European (Italy) | 23 | 3 | 13.04(4.27-33.55) | 2 | 8.70(2.18-28.99) |
| Antonio Percesepe | 2001 | European (Italy) | 12 | 0 | 3.85(0.24-40.32) | 1 | 8.33(1.16-41.32) |
| Mario Scartozzi | 2002 | European (Italy) | 37 | 2 | 5.41(1.36-19.20) | 2 | 5.41(1.36-19.20) |
| Salvatore Pucciarelli | 2003 | European (Italy) | 12 | 2 | 16.67(4.20-47.72) | 2 | 16.67(4.20-47.72) |
| M. Cravo | 1999 | European (Portugal) | 8 | 2 | 25.00(6.30-62.29) | 0 | 5.56(0.34-50.47) |
| Paulo Fidalgo | 2000 | European (Portugal) | 20 | 7 | 35.00(17.68-57.44) | 3 | 15.00(4.92-37.58) |
| Minna Nystrom-Lahti | 1996 | European (Finland) | 55 | 32 | 58.18(44.88-70.39) | 2 | 3.64(0.91-13.41) |
| Lauri A. Aaltonen | 1998 | European (Finland) | 63 | 9 | 14.29(7.60-25.24) | 1 | 1.59(0.22-10.42) |
| Reijo Salovaara | 2000 | European (Finland) | 53 | 4 | 7.55(2.86-18.45) | 1 | 1.89(0.27-12.21) |
| J-M Buerstedde | 1995 | European(Switzerland) | 10 | 3 | 30.00(9.98-62.37) | 3 | 30.00(9.98-62.37) |
| Pierre Hutter | 1998 | European(Switzerland) | 24 | 10^§^ | 41.67(24.09-61.66) | — | — |
| Karl Heinimann | 1999 | European(Switzerland) | 28 | 6 | 21.43(9.96-40.21) | 4 | 14.29(5.47-32.45) |
| Janos Papp | 2007 | European (Hungary) | 36 | 8 | 22.22(11.52-38.53) | 8 | 22.22(11.52-38.53) |
| M. Spaepen | 2006 | European (Belgium) | 225 | 10 | 4.44(2.41-8.06) | 12 | 5.33(3.05-9.15) |
| N Katballe | 2002 | European (Denmark) | 41 | 4 | 9.76(3.71-23.27) | 6 | 14.63(6.73-28.96) |
| A Apessos | 2005 | European (Greek) | 9 | 2 | 22.22(5.60-57.90) | 5 | 56.56(25.13-82.32) |
| Susan M. Farrington | 1998 | European (Scotland) | 50 | 5 | 10.00(4.22-21.87) | 7 | 14.00(6.82-26.57) |
| Rebecca A. Barnetson | 2006 | European (Scotland) | 870 | 15 | 1.72(1.04-2.84) | 16 | 1.84(1.13-2.98) |
| Jacques L. Mauillon | 1996 | European (France) | 17 | 3 | 17.65(5.80-42.71) | — | — |
| Qing Wang | 1999 | European (France) | 75 | 19 | 25.33(16.78-36.34) | 7 | 9.33(4.51-18.31) |
| B Dieumegard | 2000 | European (France) | 24 | 4 | 16.67(6.40-36.91) | 3 | 12.50(4.09-32.38) |
| Trinidad Caldes | 2002 | European (Spain) | 56 | 6^§^ | 10.71(4.89-21.87) | 4 | 7.14(2.71-17.54) |
| M Palicio | 2002 | European (Spain) | 32 | 5 | 15.63(6.66-32.47) | 0 | 1.52(0.09-20.08) |
| Virgínia Pinol | 2005 | European (Spain) | 91 | 3 | 3.30(1.07-9.73) | 6 | 6.59(2.99-13.91) |
| Mef Nilbert | 2008 | European (Spain) | 1055 | 39 | 3.70(2.71-5.02) | 58 | 5.50(4.27-7.05) |
| Martin Wehner | 1997 | European (Germany) | 29 | 5 | 17.24(7.36-35.32) | 3 | 10.34(3.37-27.60) |
| C Lamberti | 1999 | European (Germany) | 96 | 15 | 15.63(9.64-24.32) | 7 | 7.29(3.52-14.51) |
| Jochen Raedle | 2001 | European (Germany) | 22 | 4 | 18.18(6.99-39.64) | 8 | 36.36(19.34-57.67) |
| Elisabeth Mangold | 2005 | European (Germany) | 454 | 93 | 20.48(17.02-24.45) | 103 | 22.69(19.07-26.77) |
| Tannergard P | 1995 | European (Sweden) | 39 | 8 | 20.51(10.60-35.96) | — | — |
| Siobhan S. Wahlberg | 1997 | European (Sweden) | 39 | 11 | 28.21(16.36-44.11) | 2 | 5.13(1.29-18.32) |
| Liu T | 1998 | European (Sweden) | 34 | 3 | 8.82(2.87-24.04) | 3 | 8.82(2.87-24.04) |
| Maria Planck | 1999 | European (Sweden) | 16 | 1 | 6.25(0.87-33.54) | 2 | 12.50(3.14-38.60) |
| Kristina Cederquist | 2004 | European (Sweden) | 23 | 5 | 21.74(9.35-42.80) | 3 | 13.04(4.27-33.55) |
| Zdena Bartosova | 2003 | European (Slovakia) | 10 | 2 | 20.00(5.04-54.07) | 2 | 20.00(5.04-54.07) |
| Metka Ravnik-Glavac | 2000 | European (Slovenia) | 29 | 3 | 10.34(3.37-27.60) | 1 | 3.45(0.48-20.79) |
| Nicholas E. Beck | 1997 | European(England) | 14 | 4 | 28.57(11.15-56.05) | 3 | 21.43(7.07-49.43) |
| N.E.Beck | 1997 | European(England) | 10 | 3 | 30.00(9.98-62.37) | 3 | 30.00(9.98-62.37) |
| I P M Tomlinson | 1997 | European(England) | 50 | 1 | 2.00(0.28-12.88) | 1 | 2.00(0.28-12.88) |
| Nicola J Froggatt | 1996 | European (England) | 17 | 3 | 17.65(5.80-42.71) | 4 | 23.53(9.12-48.55) |
| MG Coleman | 2001 | European (England) | 6 | 3 | 50.00(16.79-83.21) | 1 | 16.67(2.28-63.13) |
| C.F. Taylor | 2003 | European(England) | 111 | 27 | 24.32(17.24-33.15) | 14 | 12.61(7.61-20.18) |
| Kurzawski G | 2006 | European (Poland) | 226 | 41 | 18.14(13.64-23.71) | 37 | 16.37(12.10-21.78) |
| Yael Goldberg | 2008 | European (Israeli) | 23 | 3 | 13.04(4.27-33.55) | 10 | 43.48(25.22-63.69) |
| Yuri K. Maliaka | 1996 | European( mixed states) | 11 | 2 | 18.18(4.58-50.70) | 4 | 36.36(14.33-66.12) |
| Qing Wang | 1997 | European( mixed states) | 19 | 5 | 26.32(11.40-49.79) | 1 | 5.26(0.74-29.39) |
| Ying Wu | 1997 | European( mixed states) | 33 | 1 | 3.03(0.43-18.61) | 1 | 3.03(0.43-18.61) |
| Juul Wijnen | 1997 | European( mixed states) | 125 | 20 | 16.00(10.56-23.50) | 16 | 12.80(7.99-19.88) |
| Juul Wijnen | 1998 | European( mixed states) | 137 | — | — | 8 | 5.84(2.95-11.24) |
| Bharati V. Bapat | 1999 | European( mixed states) | 33 | 3 | 9.09(2.96-24.68) | 10 | 30.30(17.15-47.74) |
| Steffen R. Pistorius | 2000 | European( mixed states) | 38 | 6 | 15.79(7.27-30.96) | 8 | 21.05(10.89-36.78) |
| H.F.A. Vasen | 2001 | European( mixed states) | 251 | 34 | 13.55(9.84-18.36) | 40 | 15.94(11.91-21.60) |
| Kristina Lagerstedt Robinson | 2007 | European( mixed states) | 112 | 31 | 27.68(20.19-36.67) | 23 | 20.54(14.04-29.01) |
| Maija Kohonen-Corish | 1996 | Oceania(Australia) | 12 | 4 | 33.33(13.09-62.41) | 2(6)^※^ | 33.33(8.39-73.19) |
| Rodney J. Scott | 2001 | Oceania (Australia) | 95 | 21 | 22.11(14.88-31.54) | 9 | 9.47(5.00-17.21) |
| Brigitte Wolf | 2005 | Oceania (Australia) | 43 | 9 | 20.93(11.27-35.56) | 4 | 9.30(3.54-22.30) |
| Melissa C. Southey | 2005 | Oceania (Australia) | 105 | 5 | 4.76(2.00-10.93) | 4 | 3.81(1.44-9.71) |
| Bo Liu | 1995 | mixed ethnic population | 12 | 2 | 16.67(4.20-47.72) | 3 | 25.00(8.28-55.18) |
| Gabriela Moslein | 1996 | mixed ethnic population | 46 | 9 | 19.57(10.51-33.51) | 6 | 13.04(5.98-26.13) |
| Graham Casey | 2005 | mixed ethnic population | 88 | 26 | 29.55(20.97-39.86) | 32 | 36.36(27.01-46.87) |
| Bo Liu | 1996 | mixed ethnic population | 48 | 16 | 33.33(21.53-47.68) | 15 | 31.25(19.80-45.56) |
| Wade S. Samowitz | 2001 | mixed ethnic population | 130 | 5 | 3.85(1.61-8.91) | 3 | 2.31(0.75-6.91) |
| Julie M. Cunningham | 2001 | mixed ethnic population | 51 | 2 | 3.92(0.98-14.37) | 2 | 3.92(0.98-14.37) |

§ One patient has two or more mutation point.

^ Sporadic detected cases of the two papers were extracted from the results description.

^※^ Only 6 HNPCC index families were screened for *hMSH2* gene mutation.
