# Supplementary material for: Prevalence of Pathological Germline Mutations of hMLH1 and hMSH2 Genes in Colorectal Cancer
Source: PLoS One. 2013 Mar 19;8(3):e51240. doi: 10.1371/journal.pone.0051240 (PMC3602519; doi:10.1371/journal.pone.0051240)
Supplement: Table S2 — Prevalence of mutation of hMLH1 & hMSH2 genes both detected with different detection methods. (DOC) [file pone.0051240.s002.doc]

**Table S2 Prevalence of mutation of *hMLH1 & hMSH2*** genes both detected with different detection methods

|  |  | SSCP-sequencing | | | | DHPLC-sequencing | | | | DGGE- sequencing | | | | Direct sequencing | | | |
| --- | --- | --- | --- | --- | --- | --- | --- | --- | --- | --- | --- | --- | --- | --- | --- | --- | --- |
| Ethnicity | Family history | Mutation/detection | Prevalence (%) | Component ratio | I^2^ | Mutation/detection | Prevalence (%) | Component ratio | I^2^ | Mutation/detection | Prevalence (%) | Component ratio | I^2^ | Mutation/detection | Prevalence (%) | Component ratio | I^2^ |
| Asian | AC+ | 48/130 | 37.28 | 53.93 | 34.96 | 20/57 | 36.02 | 22.47 | 0.00 | — | — | — | — | 21/53 | 39.51 | 23.60 | 7.15 |
|  | AC- | 29/187 | 17.23 | 44.62 | 48.88 | 8/52 | 15.38 | 12.31 | 0.00 | — | — | — | — | 28/64 | 46.15 | 43.08 | 61.37 |
|  | Sporadic | 5/275 | 3.36 | 50.00 | 77.24 | 5/106 | 4.72 | 50.00 | 0.00 | — | — | — | — | 0/31 | 1.56 | 0.00 | 0.00 |
|  | Subtotal | 82/592 | 22.55 | 50.00 | 82.40 | 33/215 | 15.97 | 20.12 | 41.78 | — | — | — | — | 49/148 | 38.83 | 29.88 | 69.48 |
| American multiethnic | AC+ | 20/47 | 59.03 | 22.73 | 81.03 | — | — | — | — | 37/49 | 75.51 | 42.05 | 0.00 | 31/76 | 45.08 | 35.23 | 71.38 |
|  | AC- | 4/31 | 13.39 | 28.57 | 0.00 | — | — | — | — | 4/10 | 40.00 | 28.57 | 0.00 | 6/29 | 22.59 | 42.86 | 67.58 |
|  | Sporadic | 1/17 | 5.88 | 16.67 | 0.00 | — | — | — | — | — | — | — | — | 5/43 | 14.96 | 83.33 | 0.00 |
|  | Subtotal | 25/95 | 27.42 | 23.15 | 58.98 | — | — | — | — | 41/59 | 69.49 | 37.96 | 0.00 | 42/148 | 31.06 | 38.89 | 76.75 |
| European/Australian | AC+ | 45/145 | 32.66 | 19.57 | 4.50 | 42/85 | 50.50 | 18.26 | 58.72 | 88/241 | 36.48 | 38.26 | 83.62 | 55/128 | 46.62 | 23.91 | 64.26 |
|  | AC- | 25/111 | 26.07 | 22.94 | 54.86 | 8/48 | 19.06 | 7.34 | 45.76 | 45/300 | 16.57 | 41.28 | 79.63 | 31/141 | 23.51 | 28.44 | 0.00 |
|  | Sporadic | 2/57 | 4.37 | 15.38 | 0.00 | 2/61 | 3.28 | 15.38 | 0.00 | 7/19 | 37.53 | 53.85 | 54.32 | 2/14 | 14.29 | 15.38 | 0.00 |
|  | Subtotal | 72/313 | 26.25 | 20.45 | 60.86 | 52/194 | 39.39 | 14.77 | 88.84 | 140/560 | 27.54 | 39.77 | 86.82 | 88/283 | 33.57 | 25.00 | 62.29 |
