# Supplementary material for: Prevalence of Pathological Germline Mutations of hMLH1 and hMSH2 Genes in Colorectal Cancer
Source: PLoS One. 2013 Mar 19;8(3):e51240. doi: 10.1371/journal.pone.0051240 (PMC3602519; doi:10.1371/journal.pone.0051240)
Supplement: Table S3 — Meta-regression result by different variance. (DOC) [file pone.0051240.s003.doc]

**Table S3 Meta-regression result by different variance**

| Included variance | | *P* value | Original Tau-squared | Tau-squared after regression | Explained (%) |
| --- | --- | --- | --- | --- | --- |
| Published length of year | | 0.15 | 0.84 | 0.80 | 4.76 |
| Family history | | 0.00 | 0.94 | 0.73 | 22.34 |
| Ethnicity | AC+ | 0.10 | 0.55 | 0.51 | 7.27 |
|  | AC- | 0.73 | 0.40 | 0.40 | 0.00 |
|  | Sporadic | 0.19 | 1.06 | 0.97 | 8.49 |
|  | Subtotal | 0.18 | 0.83 | 0.82 | 1.20 |
| Detection  Method | AC+ | 0.26 | 0.56 | 0.56 | 0.00 |
|  | AC- | 0.29 | 0.39 | 0.39 | 0.00 |
|  | Sporadic | 0.20 | 1.06 | 1.06 | 0.00 |
|  | Subtotal | 0.30 | 0.84 | 0.81 | 3.57 |
