# Supplementary material for: Prevalence of Pathological Germline Mutations of hMLH1 and hMSH2 Genes in Colorectal Cancer
Source: PLoS One. 2013 Mar 19;8(3):e51240. doi: 10.1371/journal.pone.0051240 (PMC3602519; doi:10.1371/journal.pone.0051240)
Supplement: Table S4 — Prevalence of hMLH1 and hMSH2 gene germline mutation by types in detail. (DOC) [file pone.0051240.s004.doc]

**Table S4 Prevalence of *hMLH1* and *hMSH2*** gene germline mutation by types in detail

|  |  | |  | *hMLH1* | | | | | *hMSH2* | | | | |
| --- | --- | --- | --- | --- | --- | --- | --- | --- | --- | --- | --- | --- | --- |
| Category | Ethnicity | | Family history | Detected cases | Mutation cases | Prevalence of Mutation (%) and 95%CI | Component Ratio (%) | I^2^ | Detected cases | Mutation cases | Prevalence of Mutation (%) and 95%CI | Component Ratio (%) | I^2^ |
| Deletion | Asian | | AC+ | 256 | 9 | 7.19(4.27-11.86) | 1.14 | 0.00 | 244 | 9 | 9.76(5.62-16.40) | 1.37 | 37.38 |
|  |  | | AC- | 313 | 7 | 4.08(2.27-7.23) | 0.88 | 0.00 | 313 | 10 | 5.94(3.41-10.15) | 1.52 | 19.44 |
|  |  | | Sporadic | 408 | 0 | 0.96(0.28-3.26) | 0.00 | 0.00 | 408 | 5 | 2.35(1.05-5.14) | 0.76 | 29.64 |
|  |  | | Not clear | 35 | 0 | 1.39(0.00-18.67) | 0.00 | 0.00 | 35 | 0 | 1.39(0.00-18.67) | 0.00 | 0.00 |
|  | American multiethnic | | AC+ | 153 | 9 | 7.81(4.30-13.78) | 1.14 | 0.00 | 153 | 10 | 8.71(4.71-15.57) | 1.52 | 30.56 |
|  |  | | AC- | 70 | 2 | 5.29(1.86-14.12) | 0.25 | 0.00 | 70 | 4 | 8.20(3.59-17.65) | 0.61 | 0.00 |
|  |  | | Sporadic | 60 | 0 | 3.92(1.14-12.64) | 0.00 | 0.00 | 60 | 0 | 3.92(1.14-12.64) | 0.00 | 0.00 |
|  |  | | Not clear | 32 | 1 | 3.12(0.44-19.11) | 0.13 | 0.00 | 32 | 4 | 12.50(4.77-28.94) | 0.61 | 0.00 |
|  | European/Australian | | AC+ | 823 | 37 | 7.07(5.35-9.30) | 4.68 | 0.00 | 852 | 43 | 7.42(5.66-9.68) | 6.53 | 0.00 |
|  |  | | AC- | 863 | 27 | 5.37(3.90-7.35) | 3.41 | 0.00 | 925 | 15 | 3.80(2.57-5.57) | 2.28 | 0.00 |
|  |  | | Sporadic | 214 | 1 | 2.40(1.35-4.24) | 0.13 | 0.00 | 213 | 3 | 4.62(2.20-9.45) | 0.46 | 0.00 |
|  |  | | Not clear | 2996 | 56 | 3.44(1.49-7.72) | 7.08 | 66.30 | 2996 | 80 | 3.05(1.70-5.43) | 12.14 | 74.03 |
|  | Mixed population | | AC+ | 131 | 9 | 6.98(3.67-12.87) | 1.14 | 0.00 | 131 | 9 | 7.86(2.39-22.89) | 1.37 | 64.27 |
|  |  | | AC- | 27 | 3 | 11.14(3.64-29.41) | 0.38 | 0.00 | 27 | 0 | 3.71(0.52-22.20) | 0.00 | 0.00 |
|  |  | | Sporadic | 36 | 0 | 4.05(0.82-17.80) | 0.00 | 0.00 | 36 | 1 | 5.37(1.34-19.08) | 0.15 | 0.00 |
|  |  | | Not clear | 212 | 3 | 1.77(0.62-4.95) | 0.38 | 0.00 | 212 | 3 | 1.69(0.55-5.11) | 0.46 | 0.00 |
|  | Subtotal | | | 6629 | 164 | 3.86(3.35-4.45) | 20.73 | 10.63 | 6707 | 196 | 4.66(4.08-5.31) | 29.74 | 39.03 |
| Insertion | Asian | AC+ | | 256 | 15 | 9.59(6.27-14.38) | 1.90 | 0.00 | 244 | 3 | 4.73(2.47-8.89) | 0.46 | 0.00 |
|  |  | AC- | | 313 | 11 | 5.75(3.43-9.47) | 1.39 | 1.70 | 313 | 2 | 4.30(2.00-9.00) | 0.30 | 26.34 |
|  |  | Sporadic | | 408 | 0 | 0.96(0.28-3.26) | 0.00 | 0.00 | 408 | 0 | 0.96(0.28-3.26) | 0.00 | 0.00 |
|  |  | Not clear | | 35 | 0 | 1.39(0.00-18.67) | 0.00 | 0.00 | 35 | 0 | 1.39(0.00-18.67) | 0.00 | 0.00 |
|  | American multiethnic | AC+ | | 153 | 6 | 6.76(3.47-12.74) | 0.76 | 0.00 | 153 | 4 | 6.50(2.90-13.95) | 0.61 | 18.03 |
|  |  | AC- | | 70 | 0 | 3.46(1.00-11.23) | 0.00 | 0.00 | 70 | 0 | 3.46(1.00-11.23) | 0.00 | 0.00 |
|  |  | Sporadic | | 60 | 0 | 3.92(1.14-12.64) | 0.00 | 0.00 | 60 | 0 | 3.92(1.14-12.64) | 0.00 | 0.00 |
|  |  | Not clear | | 32 | 0 | 1.52(0.00-20.08) | 0.00 | 0.00 | 32 | 0 | 1.52(0.00-20.08) | 0.00 | 0.00 |
|  | European/Australian | AC+ | | 823 | 13 | 5.17(3.63-7.32) | 1.64 | 0.00 | 852 | 8 | 3.55(2.42-5.20) | 1.21 | 0.00 |
|  |  | AC- | | 863 | 7 | 3.17(2.06-4.85) | 0.88 | 0.00 | 925 | 5 | 2.78(1.77-4.36) | 0.76 | 0.00 |
|  |  | Sporadic | | 214 | 2 | 4.69(2.17-9.85) | 0.25 | 0.00 | 213 | 0 | 3.44(1.49-7.72) | 0.00 | 0.00 |
|  |  | Not clear | | 2996 | 38 | 1.48(0.61-3.52) | 4.80 | 73.61 | 2996 | 21 | 1.49(0.01-2.22) | 3.19 | 45.11 |
|  | Mixed population | AC+ | | 131 | 8 | 6.36(3.21-12.21) | 1.01 | 0.00 | 131 | 5 | 4.72(2.06-10.47) | 0.76 | 0.00 |
|  |  | AC- | | 27 | 0 | 3.71(0.52-22.20) | 0.00 | 0.00 | 27 | 0 | 3.71(0.52-22.20) | 0.00 | 0.00 |
|  |  | Sporadic | | 36 | 0 | 4.05(0.82-17.80) | 0.00 | 0.00 | 36 | 1 | 5.37(1.34-19.08) | 0.15 | 0.00 |
|  |  | Not clear | | 212 | 1 | 0.97(0.24-3.80) | 0.13 | 0.00 | 212 | 0 | 0.83(0.17-4.01) | 0.00 | 0.00 |
|  | Subtotal | | | 6629 | 101 | 3.69(3.12-4.36) | 12.77 | 31.41 | 6707 | 49 | 2.18(1.75-2.70) | 7.44 | 0.00 |
| Substitution | Asian | AC+ | | 256 | 38 | 17.74(13.24-23.37) | 4.80 | 0.00 | 244 | 18 | 11.37(7.37-17.14) | 2.73 | 6.55 |
|  |  | AC- | | 313 | 25 | 12.34(8.49-17.59) | 3.16 | 49.14 | 313 | 16 | 7.37(3.27-15.78) | 2.43 | 58.22 |
|  |  | Sporadic | | 408 | 3 | 2.83(1.12-6.99) | 0.38 | 44.86 | 408 | 2 | 1.44(0.51-4.05) | 0.30 | 0.00 |
|  |  | Not clear | | 35 | 0 | 1.39(0.00-18.67) | 0.00 | 0.00 | 35 | 5 | 14.28(6.06-30.05) | 0.76 | 0.00 |
|  | American multiethnic | AC+ | | 153 | 18 | 15.10(6.52-31.21) | 2.28 | 59.82 | 153 | 4 | 6.56(3.03-13.61) | 0.61 | 0.00 |
|  |  | AC- | | 70 | 4 | 11.77(5.01-25.21) | 0.51 | 35.00 | 70 | 0 | 3.46(1.00-11.23) | 0.00 | 0.00 |
|  |  | Sporadic | | 60 | 4 | 10.28(4.28-22.70) | 0.51 | 0.00 | 60 | 2 | 5.89(2.08-15.61) | 0.30 | 0.00 |
|  |  | Not clear | | 32 | 3 | 9.38(3.06-25.35) | 0.38 | 0.00 | 32 | 5 | 15.63(6.66-32.47) | 0.76 | 0.00 |
|  | European/Australian | AC+ | | 823 | 111 | 16.54(13.87-19.60) | 14.03 | 32.32 | 852 | 64 | 11.63(9.27-14.48) | 9.71 | 23.85 |
|  |  | AC- | | 863 | 65 | 10.69(8.53-13.31) | 8.22 | 0.00 | 925 | 34 | 6.21(4.59-8.34) | 5.16 | 0.00 |
|  |  | Sporadic | | 214 | 5 | 5.94(3.02-11.35) | 0.63 | 0.00 | 213 | 5 | 5.74(2.93-10.94) | 0.76 | 0.00 |
|  |  | Not clear | | 2996 | 109 | 4.75(2.65-8.39) | 13.78 | 85.47 | 2996 | 103 | 3.24(1.64-6.32) | 15.63 | 86.16 |
|  | Mixed population | AC+ | | 131 | 18 | 14.61(9.38-22.05) | 2.28 | 28.26 | 131 | 16 | 12.69(7.91-19.73) | 2.43 | 0.00 |
|  |  | AC- | | 27 | 1 | 5.36(1.08-22.68) | 0.13 | 0.00 | 27 | 3 | 14.28(4.54-36.83) | 0.46 | 46.35 |
|  |  | Sporadic | | 36 | 4 | 11.30(4.30-26.54) | 0.51 | 0.00 | 36 | 2 | 10.28(3.30-27.76) | 0.30 | 0.00 |
|  |  | Not clear | | 212 | 6 | 3.37(1.52-7.31) | 0.76 | 8.54 | 212 | 6 | 3.43(0.93-11.89) | 0.91 | 58.66 |
|  | Subtotal | | | 6629 | 414 | 9.14(7.56-11.00) | 52.34 | 68.68 | 6707 | 285 | 6.07(4.88-7.53) | 43.25 | 63.38 |
| Large genomic rearrangement | Asian | AC+ | | 256 | 2 | 4.50(2.34-8.48) | 0.25 | 0.00 | 244 | 2 | 4.43(2.31-8.32) | 0.30 | 0.00 |
|  |  | AC- | | 313 | 3 | 5.46(2.67-10.85) | 0.38 | 38.31 | 313 | 4 | 3.79(1.94-7.27) | 0.61 | 0.00 |
|  |  | Sporadic | | 408 | 2 | 2.42(0.90-6.32) | 0.25 | 42.04 | 408 | 2 | 1.47(0.55-3.86) | 0.30 | 0.00 |
|  |  | Not clear | | 35 | 0 | 1.39(0.00-18.67) | 0.00 | 0.00 | 35 | 0 | 1.39(0.00-18.67) | 0.00 | 0.00 |
|  | American multiethnic | AC+ | | 153 | 2 | 3.79(1.58-8.80) | 0.25 | 0.00 | 153 | 12 | 16.19(9.87-25.41) | 1.82 | 49.64 |
|  |  | AC- | | 70 | 0 | 3.46(1.00-11.23) | 0.00 | 0.00 | 70 | 0 | 3.46(1.00-11.23) | 0.00 | 0.00 |
|  |  | Sporadic | | 60 | 0 | 3.92(1.14-12.64) | 0.00 | 0.00 | 60 | 0 | 3.92(1.14-12.64) | 0.00 | 0.00 |
|  |  | Not clear | | 32 | 0 | 1.52(0.00-20.08) | 0.00 | 0.00 | 32 | 0 | 1.52(0.00-20.08) | 0.00 | 0.00 |
|  | European/Australian | AC+ | | 823 | 39 | 11.15(8.51-14.48) | 4.93 | 45.49 | 852 | 16 | 5.17(3.70-7.19) | 2.43 | 0.00 |
|  |  | AC- | | 863 | 14 | 4.40(3.04-6.32) | 1.77 | 0.00 | 925 | 17 | 3.89(2.72-5.54) | 2.58 | 0.00 |
|  |  | Sporadic | | 214 | 1 | 3.43(1.54-7.44) | 0.13 | 0.00 | 213 | 2 | 5.62(2.60-11.70) | 0.30 | 35.56 |
|  |  | Not clear | | 2996 | 23 | 1.32(0.55-3.17) | 2.91 | 69.36 | 2996 | 36 | 1.93(1.42-2.62) | 5.46 | 15.19 |
|  | Mixed population | AC+ | | 131 | 8 | 5.03(0.91-23.51) | 1.01 | 66.05 | 131 | 14 | 11.97(7.27-19.07) | 2.12 | 0.00 |
|  |  | AC- | | 27 | 0 | 3.71(5.19-22.20) | 0.00 | 0.00 | 27 | 2 | 10.80(1.03-58.57) | 0.30 | 58.69 |
|  |  | Sporadic | | 36 | 2 | 6.84(1.99-20.97) | 0.25 | 0.00 | 36 | 3 | 9.71(3.44-24.50) | 0.46 | 0.00 |
|  |  | Not clear | | 212 | 0 | 0.83(0.17-4.01) | 0.00 | 0.00 | 212 | 0 | 0.83(0.17-4.01) | 0.00 | 0.00 |
|  | Subtotal | | | 6629 | 96 | 2.49(1.80-3.42) | 12.14 | 59.28 | 6707 | 110 | 3.82(3.26-4.48) | 16.69 | 42.06 |
| Not identified | Asian | AC+ | | 256 | 1 | 3.94(2.02-7.55) | 0.13 | 0.00 | 244 | 0 | 3.64(1.78-7.29) | 0.00 | 0.00 |
|  |  | AC- | | 313 | 0 | 2.50(1.09-5.68) | 0.00 | 0.00 | 313 | 0 | 2.50(1.09-5.68) | 0.00 | 0.00 |
|  |  | Sporadic | | 408 | 2 | 2.52(0.94-6.58) | 0.25 | 46.50 | 408 | 0 | 0.96(0.28-3.26) | 0.00 | 0.00 |
|  |  | Not clear | | 35 | 0 | 1.39(0.00-18.67) | 0.00 | 0.00 | 35 | 0 | 1.39(0.00-18.67) | 0.00 | 0.00 |
|  | American multiethnic | AC+ | | 153 | 6 | 8.07(4.23-14.86) | 0.76 | 0.00 | 153 | 5 | 6.87(3.45-13.19) | 0.76 | 0.00 |
|  |  | AC- | | 70 | 2 | 7.67(2.84-19.07) | 0.25 | 0.00 | 70 | 2 | 7.67(2.84-19.07) | 0.30 | 0.00 |
|  |  | Sporadic | | 60 | 0 | 3.92(1.14-12.64) | 0.00 | 0.00 | 60 | 0 | 3.92(1.14-12.64) | 0.00 | 0.00 |
|  |  | Not clear | | 32 | 0 | 1.52(0.00-20.08) | 0.00 | 0.00 | 32 | 0 | 1.52(0.00-20.08) | 0.00 | 0.00 |
|  | European/Australian | AC+ | | 823 | 4 | 4.14(2.78-6.12) | 0.51 | 0.00 | 852 | 6 | 4.43(2.99-6.52) | 0.91 | 0.00 |
|  |  | AC- | | 863 | 1 | 2.80(1.74-4.47) | 0.13 | 0.00 | 925 | 5 | 3.67(2.35-5.70) | 0.76 | 0.00 |
|  |  | Sporadic | | 214 | 0 | 3.44(1.49-7.72) | 0.00 | 0.00 | 213 | 0 | 3.44(1.49-7.72) | 0.00 | 0.00 |
|  |  | Not clear | | 2996 | 0 | 0.61(0.28-1.31) | 0.00 | 16.74 | 2996 | 1 | 0.76(0.36-1.60) | 0.15 | 36.95 |
|  | Mixed population | AC+ | | 131 | 0 | 1.24(0.25-5.90) | 0.00 | 0.00 | 131 | 0 | 1.24(0.25-5.90) | 0.00 | 0.00 |
|  |  | AC- | | 27 | 0 | 3.71(0.52-22.20) | 0.00 | 0.00 | 27 | 0 | 3.71(0.52-22.30) | 0.00 | 0.00 |
|  |  | Sporadic | | 36 | 0 | 4.05(0.82-17.80) | 0.00 | 0.00 | 36 | 0 | 4.05(0.82-17.80) | 0.00 | 0.00 |
|  |  | Not clear | | 212 | 0 | 0.83(0.17-4.01) | 0.00 | 0.00 | 212 | 0 | 0.83(0.17-4.01) | 0.00 | 0.00 |
|  | Subtotal | | | 6629 | 16 | 2.30(1.79-2.95) | 2.02 | 4.34 | 6707 | 19 | 2.57(2.00-3.29) | 2.88 | 28.95 |
