# Supplementary material for: Prevalence of Pathological Germline Mutations of hMLH1 and hMSH2 Genes in Colorectal Cancer
Source: PLoS One. 2013 Mar 19;8(3):e51240. doi: 10.1371/journal.pone.0051240 (PMC3602519; doi:10.1371/journal.pone.0051240)
Supplement: Table S5 — Prevalence of hMLH1 and hMSH2 gene germline mutation by clinic and population-based. (DOC) [file pone.0051240.s005.doc]

**Table S5 Prevalence of *hMLH1* and *hMSH2* gene germline mutation by** clinic and population-based

|  |  | *hMLH1* | | | | | *hMSH2* | | | | |
| --- | --- | --- | --- | --- | --- | --- | --- | --- | --- | --- | --- |
| Family history | Population original | Detected cases | Mutation cases | Prevalence of Mutation (%) and 95%CI | I^2^ | Difference between subgroups | Detected cases | Mutation cases | Prevalence of Mutation (%) and 95%CI | I^2^ | Difference between subgroups |
| AC+ | Population-based | 449 | 125 | 29.30(25.04-33.94) | 41.11 | *P* =0.71 | 449 | 100 | 21.83(15.11-30.47) | 64.43 | *P* =0.88 |
|  | Clinic-based | 316 | 90 | 30.65(25.38-36.48) | 45.07 |  | 263 | 53 | 23.83(18.56-30.05) | 31.92 |  |
| AC- | Population-based | 337 | 29 | 10.37(7.34-14.47) | 0.00 | *P* =0.05 | 337 | 26 | 10.06(6.87-14.50) | 48.45 | *P* =0.37 |
|  | Clinic-based | 231 | 36 | 18.28(13.51-24.25) | 6.14 |  | 204 | 18 | 12.95(8.52-19.20) | 12.60 |  |
| Sporadic | Population-based | 227 | 7 | 6.86(3.34-13.56) | 45.03 | *P* =0.71 | 227 | 11 | 6.36(2.48-15.38) | 50.65 | *P* =0.96 |
|  | Clinic-based | 276 | 3 | 3.48(0.38-25.56) | 70.61 |  | 275 | 5 | 5.88(0.24-61.62 | 86.25 |  |
| Total^*^ | Population-based | 3539 | 289 | 12.49(8.65-17.71) | 88.54 | *P* =0.13 | 3539 | 273 | 10.50(6.94-15.59) | 89.51 | *P* =0.62 |
|  | Clinic-based | 1609 | 243 | 17.39(13.62-21.93) | 62.24 |  | 1528 | 199 | 12.03(8.47-16.80) | 70.63 |  |

* Total include family history not clear group
