# Supplementary material for: Prevalence of Pathological Germline Mutations of hMLH1 and hMSH2 Genes in Colorectal Cancer
Source: PLoS One. 2013 Mar 19;8(3):e51240. doi: 10.1371/journal.pone.0051240 (PMC3602519; doi:10.1371/journal.pone.0051240)
Supplement: Table S6 — Prevalence of hMLH1 and hMSH2 gene intron area germline mutation in different family history and ethnicity. (DOC) [file pone.0051240.s006.doc]

**Table S6 Prevalence of *hMLH1* and *hMSH2* gene intron area** germline mutation in different family history and ethnicity

|  |  | *hMLH1* | | | | | *hMSH2* | | | | |
| --- | --- | --- | --- | --- | --- | --- | --- | --- | --- | --- | --- |
| Family history | Ethnicity | Detected cases | Mutation  cases | Prevalence (%) and 95%CI | *P* for  Z test | I^2^ (%) | Detected cases | Mutation  cases | Prevalence (%) and 95%CI | *P* for  Z test | I^2^ (%) |
| AC+ | Asian | 137 | 10 | 8.62 (4.75-15.13) | 0.00 | 0.00 | 137 | 6 | 7.72(3.79-15.06) | 0.00 | 0.00 |
|  | American multiethnic | 94 | 7 | 8.26(4.09-16.00) | 0.03 | 0.00 | 94 | 3 | 3.86 (1.36-10.48) | 0.00 | 0.00 |
|  | European/ Australian | 430 | 18 | 6.80(4.43-10.31) | 0.00 | 0.00 | 414 | 12 | 4.76(2.90-7.70) | 0.00 | 0.00 |
|  | Mixed population^﹡^ | 63 | 9 | 14.29(7.60-25.24) | 0.00 | 0.00 | 63 | 4 | 6.35(2.40-15.73) | 0.00 | 0.00 |
|  | Subtotal | 724 | 44 | 8.49(6.43-11.13) | 0.00 | 0.00 | 708 | 25 | 5.42(3.82-7.64) | 0.00 | 0.00 |
| AC- | Asian | 210 | 1 | 2.19(0.77-6.10) | 0.00 | 0.00 | 210 | 2 | 5.19(2.02-12.71) | 0.00 | 44.12 |
|  | American multiethnic | 39 | 2 | 10.64(3.38-28.83) | 0.00 | 17.72 | 39 | 1 | 4.89(1.23-17.56) | 0.00 | 0.00 |
|  | European/ Australian | 566 | 10 | 3.77(2.28-6.20) | 0.00 | 0.00 | 542 | 5 | 2.64(1.47-4.71) | 0.00 | 0.00 |
|  | Mixed population^﹡^ | 8 | 1 | 12.50(1.73-53.73) | 0.07 | 0.00 | 8 | 2 | 25.00(6.30-62.29) | 0.34 | 0.00 |
|  | Subtotal | 823 | 14 | 4.15(2.75-6.23) | 0.00 | 0.00 | 799 | 10 | 4.01(2.57-6.21) | 0.00 | 0.00 |
| Sporadic colorectal cancer | Asian | 51 | 0 | 1.89(0.27-12.23) | 0.00 | 0.00 | 51 | 0 | 1.89(0.27-12.23) | 0.00 | 0.00 |
|  | American multiethnic | 21 | 2 | 9.61(2.41-31.38) | 0.00 | 0.00 | 21 | 1 | 7.01(1.41-28.43) | 0.00 | 0.00 |
|  | European/ Australian | 110 | 2 | 4.30(1.62-10.95) | 0.00 | 0.00 | 109 | 1 | 4.47(1.57-12.07) | 0.00 | 0.00 |
|  | Mixed population^﹡^ | 17 | 2 | 11.76(2.96-36.83) | 0.00 | 0.00 | 17 | 2 | 11.76(2.96-36.83) | 0.01 | 0.00 |
|  | Subtotal | 199 | 6 | 5.81(3.04-10.84) | 0.00 | 0.00 | 198 | 4 | 5.51(2.76-10.68) | 0.00 | 0.00 |
| Family history not clear | Asian | - | - | - | - | - | - | - | - | - | - |
|  | American multiethnic | 32 | 3 | 9.38(3.06-25.35) | 0.00 | 0.00 | 32 | 0 | 1.52(0.09-20.08) | 0.00 | 0.00 |
|  | European/ Australian | 1770 | 40 | 3.33(1.10-9.68) | 0.00 | 86.91 | 1770 | 41 | 1.98 (0.58-6.60) | 0.00 | 85.99 |
|  | Mixed population^﹡^ | 51 | 2 | 3.92(0.98-14.37) | 0.00 | 0.00 | 51 | 1 | 1.96(0.28-12.65) | 0.00 | 0.00 |
|  | Subtotal | 1853 | 45 | 3.94(1.67-9.01) | 0.00 | 82.39 | 1853 | 42 | 1.94(0.68-5.40) | 0.00 | 80.95 |
| Asian Subtotal | | 398 | 11 | 3.37(1.89-5.92) | 0.00 | 0.00 | 398 | 8 | 3.92(2.04-7.39) | 0.00 | 19.07 |
| American multiethnic Subtotal | | 186 | 14 | 8.31(4.96-13.61) | 0.00 | 0.68 | 186 | 5 | 3.39(1.53-7.35) | 0.00 | 0.00 |
| European/Australian Subtotal | | 2876 | 70 | 3.74(2.44-5.70) | 0.00 | 60.41 | 2835 | 59 | 2.40(1.46-3.92) | 0.00 | 58.24 |
| Mixed population Subtotal^﹡^ | | 139 | 14 | 8.58(2.52-25.43) | 0.00 | 66.29 | 139 | 9 | 5.56(1.32-20.58) | 0.00 | 55.31 |
| Total | | 3599 | 109 | 4.34(3.14-5.97) | 0.00 | 58.00 | 3558 | 81 | 3.93(3.19-4.83) | 0.00 | 48.24 |

﹡This type of population include American , European and Australian.
