# Supplementary material for: Prevalence of Pathological Germline Mutations of hMLH1 and hMSH2 Genes in Colorectal Cancer
Source: PLoS One. 2013 Mar 19;8(3):e51240. doi: 10.1371/journal.pone.0051240 (PMC3602519; doi:10.1371/journal.pone.0051240)
Supplement: Figure S3 — The funnel plot of prevalence of hMSH2 gene mutation in colorectal cancer. (DOC) [file pone.0051240.s009.doc]

**Figure S3** **The funnel plot of prevalence of *hMSH2* gene mutation in colorectal cancer**

**-5**

**-4**

**-3**

**-2**

**-1**

**0**

**1**

**2**

**3**

**4**

**5**

**0.0**

**0.5**

**1.0**

**1.5**

**2.0**

**Standard Error**

**Logit event rate**

**Funnel Plot of Standard Error by Logit event rate in** AC+

**-4**

**-3**

**-2**

**-1**

**0**

**1**

**2**

**3**

**4**

**0.0**

**0.5**

**1.0**

**1.5**

**2.0**

**Standard Error**

**Logit event rate**

**Funnel Plot of Standard Error by Logit event rate in** **AC-**

**-6**

**-5**

**-4**

**-3**

**-2**

**-1**

**0**

**1**

**2**

**3**

**4**

**5**

**6**

**0.0**

**0.5**

**1.0**

**1.5**

**2.0**

**Standard Error**

**Logit event rate**

**Funnel Plot of Standard Error by Logit event rate in sporadic**

**-5**

**-4**

**-3**

**-2**

**-1**

**0**

**1**

**2**

**3**

**4**

**5**

**0.0**

**0.5**

**1.0**

**1.5**

**2.0**

**Standard Error**

**Logit event rate**

**Funnel Plot of Standard Error by Logit event rate in not clear**
