# Supplementary material for: Prevalence of Pathological Germline Mutations of hMLH1 and hMSH2 Genes in Colorectal Cancer
Source: PLoS One. 2013 Mar 19;8(3):e51240. doi: 10.1371/journal.pone.0051240 (PMC3602519; doi:10.1371/journal.pone.0051240)
Supplement: Figure S4 — Process of study selection. (DOC) [file pone.0051240.s010.doc]

**Figure S4 Process of study selection**

2200 Citations identified by search

304 Duplicate studies

1896 Potentially relevant citations identified

819 Articles excluded after title review

147 Not focus on colorectal cancer

134 Associated with cancer prognosis

796 Abstracts retrieved

353 Articles excluded after abstract review

148 Not relevant to MMR gene mutation

16 Not referred to detection

279 full manuscripts retrieved

60 articles had no unambiguous objective data can be extracted

27 articles were reviews

24 articles’ data were overlapping with others

22 articles, the cases were known carried germline mutation

13 articles were case reports

13 articles about mutations in specified area or specified mutation types

4 articles only show novel mutation

4 articles about mutation nomenclature4 articles included other cancer such as endometrial carcinoma

3 articles about protein

2 articles about somatic mutation

1 article about single nucleotide polymorphism

102 papers went into this meta-analysis
